# Improving the Use of Simulation in Nursing Education: Protocol for a Realist Review

**DOI:** 10.2196/16363

**Published:** 2020-04-29

**Authors:** Torbjørg Træland Meum, Åshild Slettebø, Mariann Fossum

**Affiliations:** 1 Department of Health and Nursing Science University of Agder Grimstad Norway

**Keywords:** education, nursing, learning, realist review, simulation training

## Abstract

**Background:**

Nursing education has evolved in line with societal needs, and simulation-based learning (SBL) is increasingly being used to bridge the gap between practice and education. Previous literature reviews have demonstrated the effectiveness of using SBL in nursing education. However, there is a need to explore how and why it *works* to expand the theoretical foundation of SBL. Realist reviews are a theory-based approach to synthesizing existing evidence on how complex programs work in particular contexts or settings.

**Objective:**

This review aims to understand how, why, and in what circumstances the use of simulation affects learning as part of the bachelor’s program in nursing.

**Methods:**

A realist review will be conducted in accordance with the realist template for a systematic review. In particular, we will identify and explore the underlying assumption of how SBL is supposed to work, that is, identify and explore program theories of SBL. The review will be carried out as an iterative process of searching, appraising, and synthesizing the evidence to uncover theoretical concepts that explain the causal effects of SBL. In the final section of the review, we will involve stakeholders in the Norwegian community in a web-based Delphi survey to ensure that the emerging theoretical framework derived from the published literature aligns with stakeholders’ experience in practice.

**Results:**

The Norwegian Centre for Research Data (project number 60415) has approved the study. We have performed an initial literature search, whereas quality appraisal and data extraction are ongoing processes.

**Conclusions:**

The final outcome of the review is anticipated to extend the theoretical foundation for using simulation as an integrated component of the bachelor’s program in nursing. Furthermore, the findings will be used to produce a briefing document containing guidance for national stakeholders in the community of simulation-based nursing education. Finally, the review findings will be disseminated in a peer-reviewed journal as well as national and international conferences.

**International Registered Report Identifier (IRRID):**

DERR1-10.2196/16363

## Introduction

The health care sector has undergone radical changes over the last few decades, a fact that has also affected nursing education. Demographic changes, technological development, and innovation in health science have led to increasing requirements being placed on the education of health professionals. A study carried out by the Carnegie Foundation for the Advancement of Teaching [1] has explored the strengths and weaknesses of nursing education, and the authors call for a radical transformation of nursing education. In particular, they describe a gap between education and practice, that is, “the ability of a practice setting to adopt and reflect what was taught in academic institutions” [1]. The authors also highlight the practice-education gap “as it is becoming more and more difficult for nursing education to keep pace with the rapid changes in clinical practice driven by research and new technologies” [1]. Patient safety is the cornerstone of high-quality health care [2]. The Institute of Medicine has highlighted evidence-based practice as one of the key competences all clinicians should possess to meet the needs of 21st-century health care services [3]*.* These competences have been adapted to the nursing community, which has in turn proposed statements on the knowledge, skills, and attitudes that should be developed during prelicensure nursing education [4]. The ability to integrate scientific knowledge into practice requires good clinical judgment and reasoning, which is considered an essential part of nursing. Clinical judgment in nursing has been defined as “interpretation or conclusion about a patient’s needs, concerns, or health problems, and/or the judgement to take action (or not), use or modify standard approaches, or improvise new ones as deemed appropriate by the patient’s response” [5]. From this point of view, clinical reasoning refers to the processes by which nurses and other clinicians arrive at their judgment, including analytical processes, intuition, and narrative thinking [5]. Similar research has also pointed to the situated, practice-based aspect of clinical reasoning and emphasized the ability to discern the relevance of the evidence behind general scientific knowledge and how it applies to a particular clinical situation [6]. The rapid expansion of clinical knowledge over the past decades has led to increasing demands on health professionals to employ evidence-based practice [3]. Clinical judgment is a capability that evolves in line with clinical experience, and the challenge is to integrate clinical knowledge into classroom teaching [7].

Simulation-based learning (SBL) has in recent years been increasingly utilized to bridge the gap between practice and education. The International Nursing Association for Clinical Simulation and Learning has defined simulation as “an educational strategy in which a particular set of conditions are created or replicated to resemble authentic situations that are possible in real life” [8]. The use of simulation in the education of health professionals is not a new innovation; however, the evolving use of advanced technology in recent decades has provided new capabilities for the educational use of simulation. Some of the early versions of full-body patient simulators were designed for subject-specific areas. For example, a Norwegian company developed Resuci Anne in 1960 for cardiopulmonary resuscitation training [9,10]. Since then, full-scale patient simulators that facilitate the formation of dynamic patient situations that fully mirror actual clinical settings have become available [11]. Accordingly, a number of simulation modalities, such as full- and part-body models with low- and high-tech features, computer-based programs, and standardized patients, have been used as an educational resource in health care [11]. Since then, simulation has gradually been integrated into the education of health professionals as an educational intervention that includes digital technologies, human resources, and educational strategies. Experiential learning [12] and situated learning theory [13] have inspired the design and development of simulated learning activities in nursing [14]. These theories emphasize active engagement in the learning process, in which knowledge is created through the transformation of experience. All in all, advancement in educational principles and digital technology has provided the opportunity to create realistic learning activities in a safe environment and, thus, bridge the gap between the classroom and clinical practice.

A growing body of evidence in the simulation literature has demonstrated the effectiveness of simulation in the teaching of clinical knowledge, procedural skills, and teamwork [11,15]. A systematic literature review by Lapkin et al [16] showed that the use of patient simulation manikins in teaching improved knowledge acquisition, critical thinking, and the ability to identify deteriorating patients. Similarly, a recent literature review conducted by Cant and Copper [17] revealed positive outcomes of simulation education for knowledge acquisition, psychomotor skills, self-efficiency, satisfaction, confidence, and critical thinking. Virtual patient simulation is also increasingly used in nursing education. A randomized controlled trial by Liaw et al [18] compared virtual simulation with manikin-based simulation and demonstrated that both the simulation modalities were effective learning strategies for improving nursing students’ clinical performance. Although previous research has shown promising effects for using simulation as an educational intervention, studies have also called for a deeper theoretical understanding of how and why simulation contributes to learning [19-22].

The realist review is a method that is increasingly used in health care to explore “What works for whom, in what circumstances and why?” [23-25]. The method is based on a realist philosophy of science, which positions itself between positivism and constructivism [23]. Basically, a realist approach recognizes the existence of an external social reality. However, there is a social reality that cannot be measured directly because our knowledge of it is processed through our brains, human senses, language, and culture. The realist review was developed as a theory-based approach to synthesizing existing evidence on how complex programs work in particular contexts or settings. The unit of analyses is to identify, test, and refine the program theory or theories, that is, the underlying assumptions about how an intervention is supposed to work [24]. The analytical process in realist review emphasizes the causal relationship between contexts, mechanisms, and outcomes (CMO). This process is described as the CMO configuration where the concept of mechanism is considered as the causal force that operates in a particular context to generate the desired outcome. All in all, the analytical process involves iterative testing of the CMO configurations and refinement of the theoretical concepts derived from relevant evidence (qualitative, quantitative, and mixed method studies) [24].

Realist reviews have been used in health care as an approach to the synthesis of evidence in various disciplines such as implementation science [26] and internet-based medical education [27]. Similarly, McGaghie et al [28] used a combined critical and realist review methodology to evaluate research on simulation-based education and highlighted 12 features and best practices to promote the educational impact of using simulation. Research on SBL in nursing education is extensive; however, realist reviews have rarely been used in this research community. Accordingly, we consider a realist review to be a promising method to uncover the causal effects of SBL and address the call for more theory-driven research to promote the educational use of simulation in nursing education.

## Methods

### The Purpose of the Review

The overall purpose of this review is to gain a deeper theoretical insight into how the use of simulation affects learning to promote the educational use of simulation as an integral part of the bachelor’s program in nursing. It is part of a larger research project focusing on professional development at the department of nursing of a large regional university in Norway. Moreover, the project is affiliated with a research group at the department of nursing science (health care services, ethics, and quality) that highlights innovative and practice-based research to educate future-oriented nurses. The cocreation of knowledge is one of this university’s overarching visions as it strives to develop and implement future-oriented, varied, student-centered, and practice-based teaching and learning methods at all levels of instruction. In accordance with this vision, SBL has for many years been an established method of learning as a supplement to traditional teaching. On the basis of challenges, such as an increasing intake of students, it is anticipated that SBL will be used to an even greater extent as an integrated part of the curriculum.

### Research Aim

This review aims to identify how the use of simulation affects learning in the bachelor’s program in nursing.

### Research Question

The research conducted in this study will explore what sort of SBL “works” for whom and in what circumstances. The study will supplement and expand upon previous research in this field, exploring the following questions:

1. How does the use of SBL affect the development of clinical reasoning, clinical judgment, and skills in nursing education?
2. How are the principles of learning translated into learning activities in the simulation setting?
3. What characterizes conditions in the simulation environment that facilitate learning in the bachelor’s program in nursing?

### Objectives

The research objectives of the study are as follows:

1. To conduct a realist review to understand how, why, and in what circumstances the use of simulation affects learning as part of the bachelor’s program in nursing.
2. To identify and refine program theories for SBL.
3. To make recommendations with regard to how the potential for using simulation may be utilized in the bachelor’s program in nursing.

### Study Design

**Figure 1:**
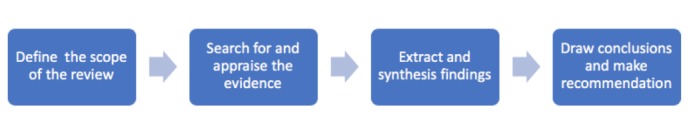
Review design.

A systematic literature review inspired by the realist approach to evaluation will be carried out [29]. The use of a realist review is a relatively new strategy for synthesizing research and methodological guidance, and training materials have been developed by an interdisciplinary research team [24]. The review process mainly follows the same steps as traditional systematic reviews. However, the unit of analysis in a realist approach is the refinement of program theory, which is characterized as an iterative process that includes both qualitative and quantitative studies [25]. Accordingly, we will utilize the realist template for systematic review [25] and follow the steps illustrated in Figure 1.

#### Stage 1: Defining the Scope of the Review

The first step is to refine the purpose of the review and identify candidate theories, that is, the theoretical basis for why SBL works [24,27]. Two researchers will take part in every step of the process, and one of the team members has extensive knowledge of SBL. On the basis of our knowledge of previous research on the use of SBL, we have acknowledged the need to explore how, why, and for whom it works. Our initial research questions will be followed with a special emphasis on *what sorts of SBL work for whom and in what circumstances.* The objective is to elucidate what and how SBL promotes clinical judgment and clinical skills in the bachelor’s program in nursing and then explain this elucidation through the use of middle-range theory. A key point at this stage is to identify existing theories regarding the use of SBL in the bachelor’s program in nursing. Previous research has identified Jeffries Framework [30], Kolb’s theory of experiential learning [12], and Bandura’s social cognitive theory [31] as the most frequently used frameworks and theories in SBL [22]. These theories are characterized as middle-range theories (theories that can usefully be applied to a family of interventions) [24]. These theories, therefore, provide a starting point in the process of identifying program theories that strive to explain the chain of events underlying SBL. As this is an iterative process, during the search and screening stages, we will attempt to expand the list of relevant middle-range theories.

#### Stage 2: Searching For and Appraising the Evidence

The search strategy will follow the guidelines provided by Booth et al [32] and include six relevant web-based databases: Cochrane Library, Cumulative Index of Nursing and Allied Health Literature, Medical Literature Analysis and Retrieval System Online, EMBASE, Education Resources Information Center, and Web of Science. The search for evidence will be carried out in collaboration with a health science librarian. Furthermore, it will mainly include the following search terms: “Nursing students,” Nursing education,” Baccalaureate,” “Simulation,” “Simulated environment,” “Simulation training,” “Manikin,” and “Anatomic model.” Each search will use the relevant search terms or MESH/thesaurus/keyword heading for each database. As indicated by our research questions, we will focus on outcomes related to clinical judgment, clinical reasoning, and clinical skills. However, we have not included these terms in our database search because of their ambiguity, which may lead to the exclusion of relevant studies. To ensure sensitivity, we will do a manual screening with these terms as part of the inclusion criteria. SBL is a comprehensive field of study, and we need to make some pragmatic decisions for which studies we want to include and exclude. Simulation in health care is usually classified in terms of fidelity, that is, the level of realism associated with a particular simulation activity [33]. However, the concept of “fidelity” is not clearly stated in the literature [34], and we will use the definitions drawn up by the Society for Simulation in Healthcare to distinguish between the physical, psychological, and environmental aspects of fidelity [33]. Recent literature reviews have mainly focused on high-fidelity simulation in laboratory settings using computerized full-body manikins [35]. Although high-fidelity simulators are considered a useful learning resource, McGaghie et al [28] have emphasized the link between educational goals and simulation tools as a key principle for the effective use of simulation. Thus, we will include medium- and low-fidelity simulators in the initial screening as we consider these to be the most appropriate in teaching basic nursing. In addition, we want to identify learning outcomes using Kirkpatrick’s 4 levels of evaluation: (1) reaction, (2) learning, (3) behavior, and (4) results [36]. Accordingly, our initial inclusion and exclusion criteria are shown in Textboxes 1 and 2.

**Inclusion criteria for this study.**

- Bachelor’s degree in nursing
- Focus on the student’s perspective
- Medium- and low-fidelity simulators
- Every phase of the simulation process included
- Clinical judgment, clinical reasoning, and clinical skills
- Learning outcome related to at least level 2 of the training evaluation described by Kirkpatrick
- Peer-reviewed research paper (qualitative, quantitative, and mixed method studies) written in English
- Published between 2014 and 2019

**Exclusion criteria for this study.**

- Continuing education (nurse practitioner, advanced nursing, and midwife)
- High-fidelity simulators, serious games, electronic learning, and virtual reality
- Comparison of different simulation methods
- Interdisciplinary simulation
- Disaster management
- Review articles and doctoral thesis

The preliminary criteria have been developed for searching and identifying middle-range theories that explain *how* and *why* SBL works (or does not work). In accordance with the recommendations for realist reviews, we will include qualitative, quantitative, and mixed method studies. However, we will not conduct snowball sampling or include “grey” literature such as reports, theses, or conference papers.

Search results will be saved as text files and downloaded into a web-based software platform (Covidence) for screening and quality appraisal. Two members of the review team will screen the title and abstract with respect to the inclusion and exclusion criteria. A full-text screening and quality assessment will then be performed based on quality appraisal criteria for realist reviews, that is, relevance (whether it can contribute to theory building and/or testing) and rigor (whether the methods used to generate the relevant data are credible and trustworthy) [24]. In addition, we will use the criteria described by Dixon-Woods et al [37] as well as the Mixed Methods Appraisal Tool [38] to aid our decision making on methodological credibility. When agreement is reached, data will be converted to a flowchart to illustrate the search and screening process as well as the final selection of included papers. References and data derived from the quality assessment will first be stored in Covidence and then transferred to a Microsoft Excel spreadsheet.

#### Stage 3: Data Extraction and Synthesizing the Results

At this stage, we will transfer both papers and bibliographical data to NVivo software (QSR International) for further data extraction and syntheses. First and foremost, the characteristics of the included studies will be listed in a table. One of the advantages of NVivo is its ability to transfer bibliographical data to a classification sheet when a library is imported from a reference management software program, for example, Mendeley. Descriptive information on the included studies (titles, authors, sources, and publication year) will, thus, be transferred to the classification sheet in NVivo, which will form the starting point for further data extraction. On the basis of previous realist reviews [39], we will add data domains to the classification sheet related to research design, educational setting, educational consequences, and outcome measures. Furthermore, we will use NVivo to code section of texts that may prove useful for constructing theory and refining theoretical concepts. Generative mechanisms (mechanism of action) are considered to be the key unit of analysis, and data coding will involve identifying the interrelations between CMO in the included studies. Data extraction and synthesis are considered to be an interwoven process where “raw data” captured in the included studies will be used to make sense of the causal relationship between CMO identified in the included studies.

Data synthesis will follow the realist review guidelines through using an interpretive approach to data synthesis [24]. To make sense of our “raw” data, we will incorporate the concepts identified in the primary studies into a higher-order theoretical structure [40]. First of all, we will uncover any semirecurring patterns of behavior (demi-regularities) that may be present in the included studies. We will then explore if our initial middle-range theories are able to explain why these demi-regularities emerge under the contexts reported in our included papers [39]. The middle-range theories will be treated as theoretical data and included in a constant comparison of key demi-regularities derived from the included studies with emerging theoretical conceptualization [24]. The main objective of the review process is to refine the program theory, which may involve several iterations that include testing and refining to progress toward a refined theoretical framework of SBL in nursing education.

#### Stage 4: Drawing Conclusions and Making Recommendations

The findings will provide theoretical and practical implications for SBL in nursing education. The inductive analysis and synthesis of data extracted from the included studies have the potential to generate theoretical concepts that explain the causal effect of SBL. Uncovering the generative mechanisms provides us with the ability to form a preliminary theoretical framework in our efforts to refine the program theory of SBL [29]. In line with similar reviews, the findings will be transformed into recommendations that can be used to inform policy makers and practitioners. The intended outcome is that practitioners will take note of the findings and implement them. Thus, we expect the review’s findings to influence the design of new programs, at this stage involving practitioners. Stakeholders’ practical knowledge will be used in the refinement of the program theory to ensure that the emerging theoretical framework derived from the published literature aligns with stakeholders’ experiences in practice.

Stakeholders will mainly be involved through using a web-based Delphi method. This is a well-known method for giving structure to group processes used to identify problems, setting goals and priorities, and identifying problems as well as solutions [41]. The method has evolved into a group-facilitating technique in health and social care. It contains an iterative multistage process designed to transform opinion into group consensus [42]. The method’s democratic, structured approach that utilizes participants’ collective knowledge is considered to be a key advantage [43], having the potential to broaden the knowledge within the nursing profession [42]. The Delphi method has been used in various settings for different purposes; in this particular case, we will apply the research-based guidelines outlined by Hasson et al [42] and Okoli and Pawlowski [44] to maintain validity and credibility in the research process. Selecting qualified experts (a panel of informed individuals) plays a key role in this process. Therefore, our preparations will include using an Excel spreadsheet as a tool to categorize and identify relevant experts, that is, knowledgeable and experienced professionals in SBL. This study will include the Norwegian community, and we will attempt to get input from a wide range of simulation experts comprising associate professors, researchers, and members of professional networks. Furthermore, we will identify relevant panelists from various organizational units such as universities, the Norwegian Nurses Organisation, and “MedSimNorge” (the national network for simulation in the health care sector). Potential participants will be identified by gatekeepers in relevant organizations, and we aim to recruit about 35 participants to the panel. Furthermore, the literature synthesis’ key findings will be transformed into statements intended as briefing material for the panelists. In accordance with the guidelines for the Delphi technique, we will carry out several rounds of online questionnaires (brainstorming, narrowing, and ranking) until consensus is reached [44]. The briefing document, as well as three or four additional questions, will be sent to the participants in the process’ first round. Subsequent rounds will involve validating, refining, and ranking the statements from previous rounds. The goal of the final phase is to reach consensus about ranking relevant statements, and the panelist will be asked to rank each potential item on a 7-point Likert scale (1=strongly disagree to 7=strongly agree). The ranking results will then be collated and inserted into an Excel spreadsheet and analyzed using nonparametric statistics (Kendall W). In addition, any free-text comments from the panelist will be analyzed thematically. The findings from the Delphi survey will be summarized, a process that involves incorporating the stakeholders' points of view into the final part of the analytical process.

The steps in the review process are overlapping and iterative, which means that the review steps can be revised throughout the process as new ideas and evidence emerge. We will, thus, follow an iterative process until theoretical saturation is achieved, that is, when new data do not provide any new theoretical insight into the emerging theory. Finally, the study will be reported in accordance with publication standards for realist review [45]. A diagram will be used to present the final program theory.

### Ethics and Dissemination

This study has been approved by the Norwegian Centre for Research Data (project number 60415). The review findings will be presented at national and international conferences, including the annual conference organized by the Society for Simulation in Europe. Finally, the findings will be disseminated in a peer-reviewed journal.

## Results

The initial search generated 4830 unique references, and after initial screening, we have included 113 studies. Data extraction and synthesis are ongoing processes, and we plan to complete a first draft of the literature review in June 2020.

## Discussion

Simulation is still a relatively new field of research, and we expect that the findings from this realist review will lead to theoretical and practical implications for the use of simulation in nursing education. Several studies have emphasized the need for conceptual and theoretical frameworks for the use of simulation [14,19], and the final outcome of this review is expected to generate theoretical concepts that may explain the causal effects of simulation as an integrated part of nursing education. The findings will then be used to produce a briefing document to guide practitioners in designing educational programs for the bachelor's degree in nursing. Although the intended audience for this review is nursing educators, we expect the findings to be relevant to other health care professionals.

In this study, we have outlined the different steps involved in a realist review, and we consider it as a promising method to unpack the complexity of SBL. The realist review method has increasingly been used across different research fields; however, it has rarely been used in simulation-based nursing education. A major advantage of the method is to move beyond the effect of using simulation toward exploration of the causal mechanisms involved in the intervention. However, there are some key issues to consider when applying the method. As previously mentioned, the realist review method is based on a realist philosophy of science [29]. Thus, it is necessary to consider the underlying ontological and epistemological assumptions of the realist philosophy to understand the methodological implications. The realist approach is regarded as a middle way between positivism and interpretivism and embraces a variety of qualitative and quantitative methods [46]. This makes it particularly appropriate for the study of SBL that incorporates aspects of both natural science and social science. A central theme of a realist approach is the power of generative mechanisms; however, it can be challenging to know how to identify and define them. To gain a broader understanding of these issues, we will incorporate recent research from other research disciplines that deal with methodological implications of critical realism [46]. We, thus, expect the study to increase the insight into methodological challenges that have been pointed out in similar research [39].

## Abbreviations

CMO: contexts, mechanisms, and outcomes
SBL: simulation-based learning
